# Lower risk of end stage renal disease in diabetic nurse

**DOI:** 10.1051/bmdcn/2017070425

**Published:** 2017-11-24

**Authors:** Cheng-Chin Pan, Hsiu-Ling Huang, Ming-Chih Chen, Chuan-Yu Kung, Pei-Tseng Kung, Wen-Yu Chou, Wen-Chen Tsai

**Affiliations:** 1 Department of Urology, Hengchun Tourism Hospital, Ministry of Health and Welfare Pingtung 946 Taiwan; 2 Department of Aged Welfare & Social Work, Toko University Chiayi 613 Taiwan; 3 Department of Health Services Administration, China Medical University Taichung 404 Taiwan; 4 Department of Orthopedics, Hengchun Tourism Hospital, Ministry of Health and Welfare Pingtung 946 Taiwan; 5 Department of Nursing, Hengchun Tourism Hospital, Ministry of Health and Welfare Pingtung 946 Taiwan; 6 Department of Healthcare Administration, Asia University Taichung 413 Taiwan

**Keywords:** Nurse with Diabetes, Dialysis, National Health Insurance, KAP, Cohort Study

## Abstract

Objectives: As professional medical caregivers, nurses have extensive medical knowledge and information than general population. However, they may use their professional knowledge and networks to seek prompt health services. In this study, we aimed to determine susceptibility of nurses with diabetes to developing end-stage renal disease requiring dialysis compared to diabetes patients in the general population.

Methods: This retrospective longitudinal study extracted data of nurses with newly diagnosed diabetes and general patients with diabetes from the National Health Insurance Database between 1998 and 2006 and follow-up to December 2009, satisfied the participant inclusion criteria was 518,058. Nurses and general population were matched with propensity score method in a 1:10 ratio. Basic characteristics and health status were similar between groups. Cox proportional hazards model was used to compare relative risks and dialysis factors between groups.

Results: Nurses were younger than general population with diabetes (42.01 years *vs*. 59.29 years) and had lower risk of dialysis (adjusted hazard ratio = 0.36, 95% confidence interval 0.16-0.81). Nurses with Diabetes Complications Severity Index (DCSI)≧3 had dialysis risk up to 83.53 times higher than that of the reference group (DCSI < 3). DCSI was the only variable determined to be a related factor affecting dialysis risk in nurses with diabetes.

Conclusions: Nurses with diabetes have lower risk of dialysis. This suggests that nurses may have more knowledge regarding chronic disease control and change their lifestyles than general diabetes patients. Results of this study may serve as a reference for developing health education.

## 1. Introduction

The data from the United States Renal Data System (USRDS) in 2013 showed that Taiwan has both a high prevalence and incidence of end-stage renal disease (ESRD).[1] Molitch et al.[2] reported that the major cause of ESRD was diabetes and that the percentage increased from 25.4% in 1990 to 39.5% in 2004. The effect of diabetes on ESRD has increased 1.6 times over 15 years, which is attributable to the high prevalence of type 2 diabetes mellitus (T2DM).

T2DM is a major chronic disease worldwide, with approximately 382 million diabetes patients aged between 20 and 79 years in 2013.[3] The International Diabetes Federation (IDF) reported that 3.8 million people die of diabetes-related diseases annually around the world.[4] Therefore, early diagnosis of diabetes and provision of comprehensive and professional health education may enhance the self-care ability and personal responsibility of patients as well as to reduce related complications and mortality rate.

There are numerous studies related to illness behaviors and health seeking, the majority of which focus on specific types of patients. The health belief model,[5] the Andersen Behavioral Model of Health Services Use,[6] the Theory of Planned Behavior,[7] and the general theory of help-seeking [8] describe the basic foundation for the determinants of various diseases. However, a limited number of studies have examined the behavior of disease-affected health providers to determine whether they are influenced by their medical knowledge.

Self-care ability is a factor in patient behavior. Patient knowledge regarding their health status affects their attitude and practice. The interaction of these three aspects – knowledge, attitude and practice – has been described as the knowledge, attitude and practice theory (KAP)[9]. This theory postulates that if people have accurate knowledge of a disease and develop an active and positive attitude, they will acquire functional self-care ability, which can directly or indirectly affect their prognosis.

As professional medical caregivers, nurses have extensive medical knowledge and different health-seeking behavior and information compared to the general population.[10] Research has investigated nurses, with regard to job satisfaction,[11] stress,[12] daytime sleepiness and risk of disease,[13] and they have more sources of assistance than the general population. However, few studies have examined the relation between professional knowledge and chronic disease outcome with nurses as patients. Whether the advantage of health knowledge regarding their own diabetes ultimately reduces the risk of dialysis in nurses is unknown.

This study primarily investigated relative risk and factors related to the development of ESRD that required dialysis between nurses with newly diagnosed diabetes and patients with newly diagnosed diabetes in the general population. The results of this study could possibly serve as a reference for medical providers and related units in planning health promotion activities. Results of this study may serve as a reference for developing health education regarding chronic diseases.

## 2. Materials and Methods

### 2.1. Data source

This is a retrospective longitudinal study. The secondary data in the analysis was retrieved from the National Health Insurance Research Database (NHIRD) and used a specific diabetes mellitus (DM) database that included all nationwide DM patients. This database is an extensive source of health data that currently represents the majority of the population of Taiwan. The National Health Insurance (NHI) commenced in March 1995 as a mandatory public health insurance providing comprehensive coverage of medical services. Since 2012, the NHI covers up to 99.85% of the population in Taiwan.[14] The insurance coverage includes medications prescribed in outpatient, inpatient and emergency departments. The database includes all medical data of insured patients, including chronic disease coverage of diabetes and ESRD[15], which makes it an ideal source of data for the present study. Before the study analyses, all individual identification information was deleted, and personal privacy was protected. This study was approved by the Institutional Review Board (IRB) of China Medical University and Hospital (IRB Number: CMUH 20130326C).

### 2.2. Study population and sample

Study subjects were Taiwanese nurses with newly diagnosed diabetes and general patients with newly diagnosed diabetes who were diagnosed and treated between 1998 and 2006. We included people who were already nurses at the time of diagnosis with diabetes. Each patient had been observed from the time of their diagnosis until December 31, 2009. Because the majority of nurses in Taiwan are female (98.92%),[16] study subjects included only female nurses and female general patients with newly diagnosed diabetes. Finally, satisfied the participant inclusion criteria was 518,058 (Table 1).

**Table 1:** - Patient demographics before and after propensity score (PS) matching.

Variables	Before PS Matching						After PS Matching (10:1)						
	Total		General patients		Nurse		P-value	Total		General patients		Nurse		*P*-value
	N	%	N	%	N	%		N	%	N	%	N	%	
**Total patients**	**518058**	**100.00**	**516100**	**99.62**	**1958**	**0.38**		**18601**	**100.00**	**16910**	**90.91**	**1691**	**9.09**	
**Age**							**<0.001**							0.928
<25	3092	0.60	2913	0.56	179	9.14		1194	6.42	1083	6.40	111	6.56	
25-34	15361	2.97	14951	2.90	410	20.94		3214	17.28	2933	17.34	281	16.62	
35-44	53986	10.42	53436	10.35	550	28.09		5568	29.93	5069	29.98	499	29.51	
45-54	127935	24.70	127351	24.68	584	29.83		6059	32.57	5494	32.49	565	33.41	
55-64	136540	26.36	136363	26.42	177	9.04		1894	10.18	1717	10.15	177	10.47	
≥65	181144	34.97	181086	35.09	58	2.96		672	3.61	614	3.63	58	3.43	
**Average age (Mean, Std)**	59.22	13.15	59.29	13.11	42.01	12.03		44.14	11.86	44.19	11.87	43.60	11.78	
**Insured salary (NT$)**							**<0.001**							0.659
Low-income household	5010	0.97	5009	0.97	1	0.05		3	0.02	2	0.01	1	0.06	
≤17280	35033	6.77	34873	6.77	160	8.17		1826	9.82	1670	9.88	156	9.23	
17281~22800	295781	57.20	295390	57.34	391	19.97		4312	23.18	3922	23.19	390	23.06	
22801~28800	77247	14.94	77044	14.96	203	10.37		2011	10.81	1819	10.76	192	11.35	
28801~36300	30974	5.99	30745	5.97	229	11.70		2015	10.83	1829	10.82	186	11.00	
36301~45800	33977	6.57	33470	6.50	507	25.89		4128	22.19	3739	22.11	389	23.00	
45801~57800	19896	3.85	19584	3.80	312	15.93		2540	13.66	2311	13.67	229	13.54	
≥57801	19206	3.71	19051	3.70	155	7.92		1766	9.49	1618	9.57	148	8.75	
Missing data	934	934						
**Urbanization of residence**							**<0.001**							0.181
Level 1	141455	27.35	140779	27.33	676	34.53		6593	35.44	5998	35.47	595	35.19	
Level 2 & 3	231299	44.73	230365	44.72	934	47.70		9149	49.19	8334	49.28	815	48.20	
Level 4 & 5	94836	18.34	94589	18.36	247	12.61		2145	11.53	1944	11.50	201	11.89	
Level 6 & 7	49533	9.58	49432	9.60	101	5.16		714	3.84	634	3.75	80	4.73	
Missing data	935	935						
**Other catastrophic illnesses**							0.318							0.158
No	503285	97.15	501375	97.15	1910	97.55		18200	97.84	16554	97.89	1646	97.34	
Yes	14773	2.85	14725	2.85	48	2.45		401	2.16	356	2.11	45	2.66	
**Moderate to severe kidney disease**						0.218								0.085
No	440556	85.04	438871	85.04	1685	86.06		16425	88.30	14954	88.43	1471	86.99	
Yes	77502	14.96	77229	14.96	273	13.94		2176	11.70	1956	11.57	220	13.01	
**CCI**							**<0.001**							0.247
0	8776	1.69	8751	1.70	25	1.28		233	1.25	212	1.25	21	1.24	
1~3	129044	24.91	128410	24.88	634	32.38		6438	34.61	5881	34.78	557	32.94	
4~6	148079	28.58	147421	28.56	658	33.61		6130	32.96	5581	33.00	549	32.47	
7~9	125612	24.25	125197	24.26	415	21.20		3858	20.74	3493	20.66	365	21.58	
≥10	106547	20.57	106321	20.60	226	11.54		1942	10.44	1743	10.31	199	11.77	
**Average CCI (Mean, Std)**	6.33	3.78	6.33	3.78	5.41	3.39		5.18	3.29	5.15	3.28	5.43	3.42	
**DCSI**							**<0.001**							0.438
0	359786	69.45	358301	69.42	1485	75.84		14358	77.19	13075	77.32	1283	75.87	
1	79434	15.33	79144	15.34	290	14.81		2737	14.71	2480	14.67	257	15.20	
2	52368	10.11	52232	10.12	136	6.95		1118	6.01	1009	5.97	109	6.45	
≥3	26470	5.11	26423	5.12	47	2.40		388	2.09	346	2.05	42	2.48	
**Average DCSI (Mean, Std)**	0.54	0.98	0.54	0.98	0.37	0.76		0.34	0.73	0.34	0.73	0.36	0.75	

CCI, Charlson Comorbidity Index; DCSI, Diabetes Complications Severity Index; PS, propensity score. It’s 32 New Taiwan Dollar (NT$) per US dollar. Urbanization level of residence area (overall 7 levels; Level 1 was the most urbanized). The boldface indicated that p values less than 0.05 are considered statistically significant.

The number of nurses with newly diagnosed diabetes was less than the number of general patients with diabetes; this study used propensity score matching (PSM) with a 1:10 ratio (nurses: general patients) for objective analysis of the risks of dialysis. For comparison, the PSM is applied widely in the health care field,[17] to account for selection bias and obtain better participation effects on outcome compliance.

This result was calculated by logistic regression using the 7 covariates listed in Table 1. The majority of the variables were significantly different between the two groups. After propensity score matching, no significant differences were found in any variable between the two groups (*P* > 0.05, Table 1). Finally, the data of 18,601 study subjects were analyzed (nurses, n = 1691; general patients, n = 16,910).

### 2.3. Study design

This study defined diabetes as having diagnosis of diabetes (ICD-9-CM: 250 or A-code: A181) with three or more outpatient visits or one hospital admission within the past 365 days.[18] Patients with type 1 diabetes, gestational diabetes, neonatal diabetes and impaired glucose tolerance (ICD-9-CM: 6488, 7751, 7902, 6480) were excluded. Patients requiring dialysis within 90 days of their diabetes diagnosis were excluded. Patients younger than 20 years and older than 90 years were excluded. Dialysis was defined as follows: patients began dialysis treatments for more than three consecutive months after a new diagnosis of diabetes between 1998 and 2006.

Nurses were part of the registry for medical personnel from the NHI from 1998 to December 31, 2009. Patients who received nursing licenses after their diagnosis of diabetes were excluded. A general patient was defined as a patient who had not registered as a licensed medical professional, such as physicians, dentists, physical therapists, and nutritionists, before December 31, 2009.

The presence of other catastrophic illnesses were defined by the National Health Insurance Administration in Taiwan, including 30 categories of major illnesses (*e.g.,* stroke, hemophilia, cancer, autoimmune diseases, chronic mental illness, congenital factor disorder, congenital hypothyroidism, etc.).[19] In this study, the presence of other catastrophic illnesses was classified as yes or no. According to Deyo *et al*.,[20] the Charlson comorbidity index (CCI) involves 17 comorbidities weighted based on severity. Additionally, the definition of diabetes complication severity index (DCSI) developed by Young *et al*.[21] was used, and complications observed upon diagnosis or prior to the last day of observation were identified.

### 2.4. Statistical analysis

This study used descriptive statistics to analyze the demographic characteristics, including age, insured salary, urbanization level of residence area (overall 7 levels; Level 1 was the most urbanized),[22] nurse status, comorbidity factors, CCI, and DCSI of the study population. To reduce the bias between the two groups, this study used the propensity score matching method with a ratio of 1:10 to generate matched study subjects before analyses. The *chi*-square test was used to compare the differences in ESRD requiring dialysis between the two groups of study subjects. A Cox proportional hazards model (Hazard ratio, HR) was used to compare relative risk and factors affecting dialysis. The Cox proportional hazards model was used to further analyze the related factors of nurses with newly diagnosed diabetes requiring dialysis. All the analyses were conducted using SAS 9.3 software (SAS Institute, NC, USA). In this study, the *P* values less than 0.05 were considered significant.

## 3. Results

### 3.1. Patient demographics

This study recruited nurses and general patients diagnosed with diabetes between 1998 and 2006. All subjects were observed for the relative risks of dialysis and were followed until the end of 2009. The mean follow-up time was 6.71 ± 2.61 years (nurses vs. general patients = 6.80 ± 2.60 vs. 6.70 ± 2.61).

In the recruitment period (Table 1), 518,058 female patients who were newly diagnosed with diabetes were included, among which 1,958 (0.38%) were nurses and 516,100 (99.62%) were general patients. In the two study populations, significant differences were found between age, insured salary, urbanization of residence, CCI and DCSI (P < 0.001).

The mean age of the nurses was younger than the age of the general patients (42.01 ± 12.03 years vs. 59.29 ± 13.11 years). Regarding the CCI and DCSI, the average CCI (5.41 ± 3.39) and DCSI (0.37 ± 0.76) of the nurses were lower than those of the general patients, indicating that compared with the general public diagnosed with diabetes, the nurses were healthier when newly diagnosed with diabetes (Table 1).

### 3.2. The relative risks of nurses with diabetes and general patients with diabetes requiring dialysis

**Fig. 1:**
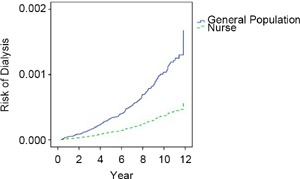
- The relative risk of requiring dialysis between nurses with diabetes and general patients with diabetes (Cox proportional hazards model was used to control for age, monthly salary, urbanization of residence, other catastrophic illnesses, CCI and DCSI.)

Bivariate analysis after matching the two groups requiring dialysis suggested that the dialysis rate of nurses was lower than that of the general patients (0.35% vs. 1.11%) (Table 2) and the difference reached statistical significance (P < 0.05). After controlling for other factors, Cox proportional hazards models were used to identify the mortality rate for the nurses and general patients. The results in Table 3 and Fig. 1 show that nurses had a lower risk of dialysis than the general public (reference group) [adjusted hazard ratio(AHR)= 0.36, 95% confidence interval (CI) 0.16-0.81]. As shown in Table 3, patients with a higher CCI had a higher risk of dialysis compared to that of the reference group (CCI ≤ 3); when the CCI increased to ≥ 5, the risk of dialysis increased up to 5.64 times (95% CI 1.79-17.75). The DCSI demonstrated similar results; patients with a higher DCSI had a higher risk of dialysis compared to that of the reference group (DCSI = 0); when the DCSI increased to ≥ 3, the risk of dialysis increased up to 400.55 times (95% CI 55.72-2879.64).

**Table 2:** - Bivariate analysis of nurses with diabetes and general patients with diabetes requiring dialysis.

Variables	Total		Without Dialysis		With Dialysis		*P*-value
	N	%	N	%	N	%	
**Total patients**	18601	100.00	18408	98.96	193	1.04
**Nurses or general patients**							**0.005**
General	16910	90.91	16723	98.89	187	1.11
Nurses	1691	9.09	1685	99.65	6	0.35
**Age**							0.247
<25	1194	6.42	1185	99.25	9	0.75
25-34	3214	17.28	3191	99.28	23	0.72
35-44	5568	29.93	5503	98.83	65	1.17
45-54	6059	32.57	5996	98.96	63	1.04
55-64	1894	10.18	1870	98.73	24	1.27
≥65	672	3.61	663	98.66	9	1.34
**Average age (Mean, Std)**	44.14	11.86	44.12	11.86	46.05	11.81
**Insured salary(NT$)**							**<0.001**
≤17280	1829	9.83	1810	98.96	19	1.04
17281~22800	4312	23.18	4258	98.75	54	1.25
22801~28800	2011	10.81	1971	98.01	40	1.99
28801~36300	2015	10.83	1997	99.11	18	0.89
36301~45800	4128	22.19	4088	99.03	40	0.97
45801~57800	2540	13.66	2527	99.49	13	0.51
≥57801	1766	9.49	1757	99.49	9	0.51
**Urbanization of residence**							0.135
Level 1	6593	35.44	6537	99.15	56	0.85
Level 2 & 3	9149	49.19	9043	98.84	106	1.16
Level 4 & 5	2145	11.53	2125	99.07	20	0.93
Level 6 & 7	714	3.84	703	98.46	11	1.54
**Other catastrophic illnesses**							0.937
No	17708	95.20	17525	98.97	183	1.03
Yes	893	4.80	883	98.88	10	1.12
**Moderate to severe kidney disease**							**<0.001**
No	15240	81.93	15233	99.95	7	0.05
Yes	3361	18.07	3175	94.47	186	5.53
**CCI**							**<0.001**
≤3	4100	22.04	4097	99.93	3	0.07
4	1928	10.37	1919	99.53	9	0.47
≥5	12573	67.59	12392	98.56	181	1.44
**Average CCI (Mean, Std)**	6.53	3.59	6.51	3.58	8.71	3.23
**DCSI**							**<0.001**
0	8687	46.70	8686	99.99	1	0.01
1	4966	26.70	4964	99.96	2	0.04
2	2730	14.68	2688	98.46	42	1.54
≥3	2218	11.92	2070	93.33	148	6.67
**Average DCSI (Mean, Std)**	1.01	1.29	0.98	1.25	3.97	1.65

CCI, Charlson Comorbidity Index; DCSI, Diabetes Complications Severity Index.

It’s 32 New Taiwan Dollar (NT$) per US dollar.

Urbanization level of residence area (overall 7 levels; Level 1 was the most urbanized).

The boldface indicated that p values less than 0.05 are considered statistically significant.

**Table 3:** - The relative risks of nurses with diabetes and general patients with diabetes requiring dialysis.

Variables	Unadj. HR	*P*-value	Adj. HR	95% CI		*P*-value
**Nurses or general patients**					
General (reference)					
Nurses	0.31	0.005	0.36	0.16	0.81	**0.013**
**Age**					
< 25 (reference)					
25-34	0.99	0.982	0.63	0.29	1.37	0.240
35-44	1.50	0.256	0.75	0.37	1.54	0.436
45-54	1.50	0.253	0.58	0.28	1.18	0.134
55-64	1.93	0.094	0.50	0.23	1.10	0.087
≥65	2.30	0.077	0.37	0.14	0.94	**0.037**
**Insured salary (NT$)**					
≤17280 (reference)					
17281~22800	1.51	0.121	1.44	0.84	2.45	0.182
22801~28800	1.52	0.132	1.24	0.71	2.15	0.447
28801~36300	0.78	0.445	0.66	0.35	1.27	0.213
36301~45800	0.83	0.496	0.77	0.44	1.33	0.350
45801~57800	0.44	0.023	0.45	0.22	0.91	**0.026**
≥57801	0.52	0.107	0.57	0.25	1.26	0.164
**Urbanization of residence**					
Level 1 (reference)					
Level 2 & 3	1.35	0.069	1.09	0.79	1.51	0.605
Level 4 & 5	1.01	0.979	0.76	0.45	1.28	0.297
Level 6 & 7	1.70	0.109	1.01	0.52	1.95	0.987
**Other catastrophic illnesses**
No (reference)
Yes	1.03	0.920	0.71	0.37	1.34	0.287
**CCI**					
(reference)					
4	5.77	0.009	3.83	1.04	14.18	**0.044**
≥5	16.45	<0.001	5.64	1.79	17.75	**0.003**
**DCSI**					
0 (reference)
1	3.26	0.334	2.94	0.27	32.42	0.380
2	117.76	<0.001	103.01	14.14	750.60	**<0.001**
≥3	475.45	<0.001	400.55	55.72	2879.64	**<0.001**

CCI, Charlson Comorbidity Index; DCSI, Diabetes Complications Severity Index;

HR, hazard ratio; CI, confidence interval.

It’s 32 New Taiwan Dollar (NT$) per US dollar.

Urbanization level of residence area (overall 7 levels; Level 1 was the most urbanized).

The boldface indicated that *p* values less than 0.05 are considered statistically significant.

### 3.3. Relative factors of nurses with diabetes requiring dialysis

As shown in Table 4, there were no significant differences between relative factors affecting nurses with diabetes requiring dialysis and those not requiring dialysis, including age, insured salary, urbanization of residence and CCI (P > 0.05); only the DCSI was significantly different (P < 0.05). If nurses had a DCSI ≥ 3, the risk of dialysis increased up to 83.53 times (95% CI 6.211123.59) that of the reference group (DCSI < 3).

**Table 4:** - Relative factors of nurses with diabetes requiring dialysis.

Variables	Unadj. HR	*P*-value	Adj. HR	95% CI		*P*-value
**Age**					
<35 (reference)					
35-44	0.72	0.747	0.25	0.02	2.96	0.269
≥45	0.54	0.538	0.16	0.01	1.99	0.154
**Insured salary(NT$)**					
≤17280 (reference)					
17281~22800	0.50	0.626	0.18	0.01	4.31	0.287
22801~28800	0.61	0.723	0.29	0.02	5.52	0.411
28801~36300	0.70	0.804	0.36	0.02	6.50	0.486
≥36301	0.36	0.403	0.18	0.01	2.34	0.188
**Urbanization of residence**					
Level 1					
Level 2 & 3	1.45	0.760	0.99	0.08	11.77	0.992
Level 4 & 5 & 6 & 7	5.58	0.137	5.37	0.51	56.81	0.163
**CCI**					
< 5 (reference)					
≥5	1.72	0.621	0.45	0.03	6.11	0.549
**DCSI**					
< 3 (reference)					
≥3	35.38	0.001	83.53	6.21	1123.59	<0.001

CCI, Charlson Comorbidity Index; DCSI, Diabetes Complications Severity Index;

HR, hazard ratio; CI, confidence interval.

It’s 32 New Taiwan Dollar (NT$) per US dollar.

Urbanization level of residence area (overall 7 levels; Level 1 was the most urbanized).

The boldface indicated that *p* values less than 0.05 are considered statistically significant.

## 5. Discussion

Because few studies have examined professional groups and their performance-related behavior, the majority of studies have used small samples or questionnaires. This is the first study using nationwide data to analyze whether the increased knowledge and resources available to nurses led to a difference in dialysis treatment between nurses and the general patients.

The results of the present study showed that the nurses were younger than the general patients (mean age 42.01 years *vs.* 59.29 years, respectively). The result was similar to that of previous studies.[23] The results suggesting that the shift work of nurses may contribute to developing diabetes at a younger age than the general public.[24] A correlation study of shift work and metabolic diseases conducted by Karlsson *et al.*[25] found that female shift workers had a higher risk of acquiring metabolic diseases than day-shift workers. However, this result may be due to screening bias; the nurses could have received the diagnosis prematurely and/or the others received it later because the awareness of the disease could be more pronounced among the nurses due to their medical knowledge. Additionally, the nurses could be more aware of the importance of preventive strategies once the disease was diagnosed, giving them a better prognosis compared to the general population. This result was similar to the conclusions in terms of their DCSI score (nursing vs. general patients = 0.37 ± 0.76 vs. 0.54 ± 0.98, Table 1).

The results in Tables 1 indicated that for the general population with newly diagnosed with diabetes aged ≧ 55 years are totally about 61.51%, but are only 12% in nurses with newly diagnosed with diabetes; this could be largely because nurses >55 year old has stopped working or stopped maintaining their licenses. This phenomenon is known as the healthy worker effect [26, 27]. Nurses must have excellent health to achieve effective performance; therefore, nurses are generally healthier than are the general population.

Because the database did not contain the related information of medication knowledge, we used propensity score matching. The nurse cohort and the general patient control group were similar in terms of demographics, health status, and their socioeconomic status (*P* > 0.05). The two groups may have differed in terms of their knowledge, attitude, and practice of health care. In the present study, the results of the bivariate analysis of nurses with diabetes and general patients with diabetes requiring dialysis found that nurses had a lower ratio of dialysis (0.35% *vs*. 1.11%) than that of patients in the general population. A Cox proportional hazards model was used to further analyze the data, and nurses had a lower risk of dialysis (AHR = 0.36) compared to general patients.

These results may be related to nurses having additional medical knowledge, as an individual’s perception of health or diseases and attitudes regarding medical care have been shown to affect health maintenance.[28] Many reports have described whether patients who received diabetes education and had self-management have significantly improved outcomes and reduced progression to dialysis.[29, 30] Nurses have a dual role of caregiver and care demander. They have additional medical care knowledge compared to the general patients, thus they may develop more positive and active disease care attitudes. They may be less sus ceptible to ESRD and have a lower risk of dialysis.

Previous studies of disease care behaviors of medical personnel and the general public found that when medical personnel sought medical care, it was based on previous personal experience with cases and personal experience seeking an informal consultation or formal treatment recommendations from colleagues. [31] After extensively collecting related information, they would then perform self-care behavior. When nurses have chronic diseases, their medical knowledge can strengthen their self-care abilities and change their lifestyles, which may result in better outcomes[32] and delay disease progression.

In the analysis of relative risk of dialysis in nurses with diabetes, only DCSI was a related factor affecting whether nurses would ultimately require dialysis. The result was similar to that of previous studies[33], which showed that the DCSI was an important determining factor of dialysis. Nurses had similar and consistent knowledge and socioeconomic status, and the DCSI was a determining factor of nurses with diabetes requiring dialysis.

### 5.1. Limitations

This study has several limitations. First, the NHIRD used the ICD-9 to define diabetes; however, these data could not be validated because no clinical data could be obtained. This study used a strict diagnosis identification, and we defined diabetes as diagnosis of diabetes (ICD-9-CM: 250 or A-code: A181) in three outpatient visits or one inpatient visit in the past 365 days, as previously described.[18] The high standard was adopted to compensate for this limitation. Second, the NHIRD did not have the related information of “lifestyle of patient,” “health behavior” and “correlation of these interventions with glycemic control,” and therefore, none of these could be included in the variables. This lack of information affected the discussion and reasoning of disease variations. However, we clearly defined the DM population and used the propensity score method to match the nurses and general population to avoid selection bias. The propensity score adjustment is an important statistical technique to reduce the bias from confounding variables in observational studies and mimic the results of a randomized controlled trial.[17] Third, because each nurse’s number of years of service and shift lengths was unknown, the correlation between their shift work and diabetes or dialysis risk could not be determined.

## 6. Conclusion

Nurses with newly diagnosed diabetes were younger than the general people with diabetes but had a lower risk of dialysis. This finding suggests that nurses may receive a more complete medical education and have additional disease care knowledge than the general public, and they may be more able to change their lifestyles. Thus, they have a lower risk of dialysis than the general population with diabetes.

Diabetes is an incurable but manageable chronic disease. The pathogenesis is complex. Regardless of whether patients are medical personnel or the general public, when they are facing the treatment of chronic disease, their self-care ability and psychological adjustment must be considered in addition to medical perspectives. An integrated care model is necessary. The results of this study could serve as a reference for developing health education programs.

## Conflicts of interest

The authors declare no conflicts of interest.
